# The spread of technological innovations: effects of psychology, culture and policy interventions

**DOI:** 10.1098/rsos.211833

**Published:** 2022-06-22

**Authors:** Denis Tverskoi, Sudarsanam Babu, Sergey Gavrilets

**Affiliations:** ^1^ National Institute for Mathematical and Biological Synthesis, University of Tennessee, Knoxville, TN 37996, USA; ^2^ Center for the Dynamics of Social Complexity, University of Tennessee, Knoxville, TN 37996, USA; ^3^ Department of Mechanical, Aerospace and Biomedical Engineering, University of Tennessee, Knoxville, TN 37996, USA; ^4^ Department of Ecology and Evolutionary Biology, University of Tennessee, Knoxville, TN 37996, USA; ^5^ Department of Mathematics, University of Tennessee, Knoxville, TN 37996, USA; ^6^ Manufacturing Science Division, Oak Ridge National Laboratory, Oak Ridge, TN 37830, USA

**Keywords:** diffusion of technological innovations, conformity, social norms, cognitive dissonance, dynamics of attitudes and beliefs

## Abstract

Technological innovations drive the evolution of human societies. The success of innovations depends not only on their actual benefits but also on how potential adopters perceive them and how their beliefs are affected by their social and cultural environment. To deepen our understanding of socio-psychological processes affecting the new technology spread, we model the joint dynamics of three interlinked processes: individual learning and mastering the new technology, changes in individual attitudes towards it, and changes in individual adoption decisions. We assume that the new technology can potentially lead to a higher benefit but achieving it requires learning. We posit that individual decision-making process as well as their attitudes are affected by cognitive dissonance and conformity with peers and an external authority. Individuals vary in different psychological characteristics and in their attitudes. We investigate both transient dynamics and long-term equilibria observed in our model. We show that early adopters are usually individuals who are characterized by low cognitive dissonance and low conformity with peers but are sensitive to the effort of an external authority promoting the innovation. We examine the effectiveness of five different intervention strategies aiming to promote the diffusion of a new technology: training individuals, providing subsidies for early adopters, increasing the visibility of peer actions, simplifying the exchange of opinions between people, and increasing the effort of an external authority. We also discuss the effects of culture on the spread of innovations. Finally, we demonstrate that neglecting the cognitive forces and the dynamic nature of individual attitudes can lead to wrong conclusions about adoption of innovations. Our results can be useful in developing more efficient policies aiming to promote the spread of new technologies in different societies, cultures and countries.

## 1. Introduction

Diffusion of technological innovations has been a major force of productivity growth throughout human prehistory and history [1,2]. For example, the spread of agriculture and innovative warfare technologies has promoted the emergence of complex societies [3,4]. It has been argued that technology diffusion has controlled the evolution of the world’s cross-country income distribution [5,6], that it can explain the structure of the world after World War II [7], and that it is a primary contributor to trade cycles [8,9]. The spread of technological innovations such as cell phones, digital television, personal computers, online banking and the Internet is changing our daily lives [10,11].

History shows though that even highly beneficial innovations can fail to spread [12,13]. Therefore, understanding how innovations spread is of great importance for businesses, governments and policy makers. Consequently, the process of the diffusion of technological innovations has been studied intensively in different fields including economics [14], marketing [15], political science [16–18], sociology [19] and anthropology [20].

Mathematical modelling has been widely used to explain and predict the diffusion of innovations starting with the seminal work of Bass [21]. Different approaches are used. Aggregate models typically use differential equations to describe the dynamics of the overall number of adopters of a new technology [22–24]. Although this approach provides a simple and often analytically tractable way for studying aggregate dynamics, it does not capture the process of individual decision-making, differences between individuals related to it, the structure and the intensity of connections between individuals, and their personal attitudes and beliefs. These limitations can be overcome with agent-based modelling (ABM) which explicitly focuses on the decision-making process and on social influences experienced by individuals [10,22,25]. In this approach, the macro-level dynamics emerge from aggregated behaviours of individuals. The vast majority of the ABM-related literature on the innovation diffusion focuses on the influence of the topological structure of social networks [26–29] including the role of social hubs [30] or the degree of the network connectivity [31] on the spread of innovations. There is also plenty of work investigating the effects of opinion leaders, word-of-mouth peer influence and social contagions [32,33].

While the social network structure is important, there are many other crucial psychological, cultural and cognitive aspects of human decision-making affecting the spread of innovations. For example, incorrect beliefs about the health and environmental effects of traditional stoves, which use wood, agricultural waste, coal and dried cattle manure, relative to those of improved cooking stoves have greatly reduced the spread of the latter in rural India [34]. Initial attempts to introduce piped drinking water made in 1950s in Uttar Pradesh, India, failed because potential adopters had negative attitudes towards the new technology due to perceived harmful effects of drinking electrically pumped water, tastelessness of this water and fears that the water was medicated to reduce fertility [35,36]. In the US home VCR market during the 1980s, the VHS format won competition over Betamax because consumers developed beliefs about its growth advantage [37], which became a ‘self-fulfilling prophecy’ [38]. It is also well recognized that social influences and social norms can play a crucial role in the innovation diffusion processes [39–43]. There are two general types of social norms [44–47]. Descriptive norms describe the perceptions of how others are behaving, such as whether one perceives their neighbours to be adopting the innovative technology [45], while injunctive norms consider the perceived expectations from others regarding a behaviour, such as feedback giving social approval for using the new technology [45]. Injunctive norms are sustained by the threat of social disapproval/punishment for norm violations and/or by norm internalization [44,48]. The literature on the innovation diffusion typically focuses on the effect of conformity and descriptive norms [49,50] while largely ignoring the effect of injunctive norms (see [51] for a rare exception).

Social norms imply conformity with behaviour and attitudes of others. Injunctive norms also include the second-order beliefs about others. Attitudes and beliefs of individuals can change in time as they interact and accumulate information about the product and the behaviour and attitudes of others as in the case of the three-stone stove in some African communities [35]. Therefore, accounting for the dynamics of beliefs can lead to a better understanding of the innovation diffusion. Modelling the dynamics of beliefs necessitates the consideration of cognitive dissonance induced by a mismatch between actions and beliefs which promotes negative emotions and discomfort [52]. To reduce the dissonance between attitudes and actions, individuals can change their attitudes via a post-factum justification of a product purchase or a technology use, or alternatively they can change their behaviour via withdrawal of the action causing the dissonance [52,53]. While some experiments studied the adoption of a new technology in situations with a mismatch between its actual performance and attitudes towards it [54–56], this phenomenon apparently has not been included yet in the innovation diffusion models.

The cultural and psychological factors just discussed have been repeatedly demonstrated to be important in decision-making [57–63]. There is a suite of corresponding mathematical models developed in economics and cultural evolution. For example, the effects of personal attitudes have been included in models of utility functions [64–68] some of which allowed for personal attitudes to change as a result of cognitive dissonance [64,65,69]. Such changes are often described by linear equations similar to those used in models of the spread of opinions [70–73]. The effects of injunctive norms and expected disapproval (or punishment) by peers and/or external authorities have been modelled by introducing additional components to the utility function [48,74–77].

However, these theoretical advances have been largely neglected in mathematical models of innovation diffusion. Here we seek to remove these limitations. Our goal will be to explore the effects of individual variation, culture, psychology and interventions on the success or failure of innovations. We will use recent advances in modelling the dynamics of actions, attitudes and beliefs of heterogeneous individuals in social dilemmas [48,76–78]. Specifically, we will build and study a model describing the joint dynamics of three important processes: individual learning and mastering a new technology, the evolution of individual attitudes towards the new technology and the evolution of individual adoption decisions. By an individual attitude, we will mean the individual perception of the desirability of its use. In our model, decisions and attitudes of individuals will affect each other through cognitive dissonance and conformity, will coevolve with existing social norms and will be subject to external influence (e.g. via advertising and mass media). Moreover, we will explore the effects of heterogeneity between individuals in different psychological and normative characteristics. Including social and psychological factors will allow us to capture the role of interpersonal and inter-group differences in various cultural characteristics (such as the degrees of individualism-collectivism, future orientation and rationality) in shaping innovation diffusion patterns. We will also consider and contrast the efficiency of several intervention strategies aiming to promote the diffusion of new technologies.

Below, after introducing our model in §2, we provide the results of its analysis in §3. We will first discuss analytical results on equilibria in some simple cases and then follow with a description of agent-based simulations of the general model. We will focus on the effects of various parameters on the frequency of adopters and individual attitudes. At the end, we will examine the effectiveness of different intervention strategies.

## 2. The model

**Figure 1.**
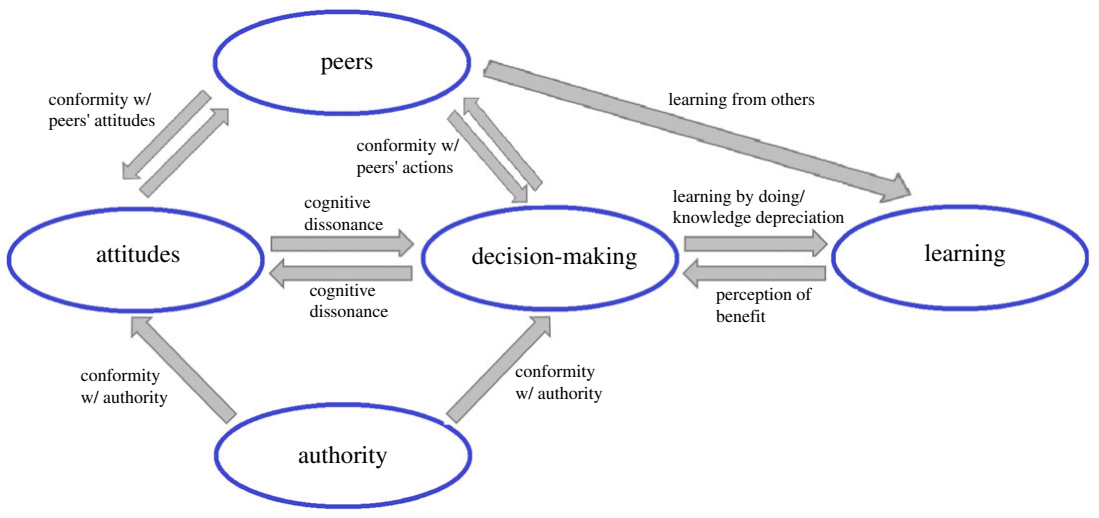
Model structure. The model integrates three interlinked processes: individual decision-making process regarding technology use, the dynamics of individual attitudes towards a new technology, and individual learning process in mastering the new technology. An individual chooses a technology by maximizing their utility function that integrates an expected material pay-off and a normative value. The expected material pay-off depends on the benefit of using a new technology, which is formed as a result of a learning process. The learning process encompasses two effects: (i) individuals can get higher benefits through repetition of an activity (i.e. learning by doing) and (ii) individuals can learn from others who use the same technology. The normative component of the utility function depends on individual attitudes (through cognitive dissonance), on the actions and attitudes of others (as a result of conformity with peers), and on the message of an external authority (as a result of conformity with the authority). The attitude of an individual, in turn, changes as a result of cognitive dissonance, conformity with peers’ actions and attitudes, and conformity with the authority.

We consider a society with *N* members manufacturing individually some product. We treat time as discrete. Each individual can use one of two technologies which we will call ‘old’ and ‘new’. The type of technology used is specified by variable *x* taking values 0 or 1 (0 means ‘old’ and 1 means ‘new’). The frequency of adopters of the new technology is specified by variable *p*. Individuals also differ in their attitude *y* towards the new technology (0 ≤ *y* ≤ 1) which affects the likelihood of its adoption and which is influenced by perceived long-term material and normative benefits, social influences (by peers and external authorities) and cognitive processes. For example, for somebody who cares mostly about material rewards or about the employment of local people, attitude *y* towards open-pit mining can be very high, whereas for people who care mostly about protecting environment it can be quite low. Similarly, even if fracking is currently not too profitable, somebody who believes it has a bright future will have high attitude *y* towards it. We assume that using the old technology results in a certain benefit. (Throughout the paper, by ‘benefit’ we understand the benefit per unit of time which can be measured in some currency.) The new technology can potentially lead to a higher benefit but achieving it requires learning. The benefit *b* of new technology depends on how long the individual has used it and on some individual characteristics. Summarizing, each individual is characterized by three dynamically changing variables: *x*, *y* and *b*. We postulate that the individual adoption process is influenced by expected material benefits, by the behaviour of peers, by the messaging of an authority promoting the new technology and by personal attitude towards it. The latter can change dynamically as a result of psychological processes and different social influences. To add further realism, we allow for variation between individuals (due to cultural and psychological differences) in certain parameters specifying how these variables change. Figure 1 illustrates the structure of our model.

### 2.1. Benefit of new technology

We assume that the benefit of using the old technology is specified by a constant *b*_0_ which is the same for all people. An individual who is just starting to use the new technology gets benefit *b*_min_ while a complete mastery of the new technology brings benefit *b*_max_. It is natural to assume that *b*_min_ ≤ *b*_0_ ≤ *b*_max_. The individual benefit of the new technology *b* can also be viewed as a measure of the skills of using it.

We postulate that two processes influence the dynamics of benefit *b*. The first process is learning by doing which leads to higher benefits through repetition of an activity [79–81]. To capture this effect we assume each use of the new technology increases benefit *b* towards *b*_max_ at a rate 0 ≤ *a* ≤ 1 specific to this individual [82,83]. Note that rate *a* can be viewed as an ‘absorptive capacity’ in terms of [84].

The second process is associated with knowledge depreciation and production breakdown [85–91]. We assume that each time step when the individual does not use the new technology, their skills decline towards *b*_min_ at rate 0 ≤ *c* ≤ 1. We describe the effects of these two processes on the benefit *b* of the new technology for a focal individual using a recurrence equation

2.1 $$b^{\prime }=\left\{ \begin{array}{cc} b+\underset{\mathrm{learning}\;\mathrm{by}\;\mathrm{doing}}{\underbrace{a\cdot (b_{\max}-b)}},\; & \text{ if }x=1\;\\ b-\underset{\mathrm{knowledge}\;\mathrm{depreciation}}{\underbrace{c\cdot (b-b_{\min})}},\; & \text{ if }x=0, \end{array}\right.$$

where the prime means the next time moment.

Below, we will consider the knowledge depreciation rate *c*, which can also be treated as the rate of loss of practical skills of using the new technology due to the lack of practice, as a constant. Although, in general, *c* can be individual-specific, for the sake of simplicity, we will assume it is the same for all individuals. In contrast, we will assume that the learning rate *a* depends on the frequency of adopters *p*

$$a=a_{0}+a_{1}p,$$

where *a*_0_ and *a*_1_ are constant individual-specific positive parameters. For example, the amount of information about the new technology available through specialized guides, forums and courses [84] can increase with *p*, which would help new users become more comfortable with the new technology increasing their rate of learning *a*. To guarantee that the learning rate *a* is between 0 and 1, we assume that *a*_0_ + *a*_1_ ≤ 1.

For an individual continuously using the new technology, equation (2.1) would generate a learning curve [85,92–96] approaching *b*_max_ asymptotically.

### 2.2. Decision-making process

We assume that individuals update their choice of technology randomly and independently with probability *ν* per time step. When this happens, they choose *x* in an attempt to maximize a utility function which combines an expected material benefit, Π(x,b), and a psychological value *V*(*x*, *y*):

2.2 U(x,y,b)=Π(x,b)+ϵV(x,y),

where ε is a parameter scaling the relative importance of normative and psychological factors in the utility function. Specifically,

$$x=\left\{ \begin{array}{cc} 1,\; & \text{if}\;\Delta U(y,b)\geq 0,\;\\ 0,\; & \text{if}\;\Delta U(y,b)<0,\; \end{array}\right.$$

where the expected difference in utilities Δ*U*(*y*, *b*) = *U*(1, *y*, *b*) − *U*(0, *y*, *b*). The latter can be written as ΔU(y,b)=ΔΠ(b)+ϵΔV(y), where the difference in pay-off between the two actions ΔΠ(b)=Π(1,b)−Π(0,b) and the corresponding difference in values Δ*V*(*y*) = *V*(1, *y*) − *V*(0, *y*).

When using agent-based simulations, we will assume that individuals use myopic best response with errors with precision parameter *λ* [97]. (If *λ* = 0, individuals make decisions completely randomly; if precision is infinity, i.e. *λ* = ∞, individuals always make the decisions maximizing their utility.)

We note that accounting for normative factors is becoming more common in modelling human decision-making [48,74,77,98–102].

#### 2.2.1. Expected material benefit

We describe the individual perception of an expected material benefit of using technology *x* given their current benefit *b* as

2.3 $$\Pi (x,b)=\left\{ \begin{array}{cc} (1-\omega )b+\omega b_{\max},\; & \text{if }x=1,\;\\ b_{0},\; & \text{if }x=0.\; \end{array}\right.$$

The equation corresponding to choosing *x* = 1 implies that when considering the material benefits of the new technology, individuals take into account not only their current benefit *b*, but also the maximum possible future benefit *b*_max_, with a foresight parameter 0 ≤ *ω* ≤ 1 being the weight of the future benefit. Our approach can be interpreted within a framework of foresight [103–105]. More generally, foresight is related to the notions of prospection [106] and inter-temporal choices when people have to trade off costs and benefits at different points in time [107,108].

#### 2.2.2. Psychological effects

We postulate that besides material benefits, individuals can also pay certain psychological costs or get certain psychological benefits as a result of their actions. First, there is a psychic cost if there is a mismatch between their action *x* and attitude *y* due to cognitive dissonance [109]. We also postulate that individuals expect disapproval from those who use a different technology and approval from those who use the same technology [110]. We assume that individuals know the average attitude $\bar{y}$ of their peers (e.g. through direct or on-line discussions) and experience additional psychological pay-off depending on whether their action aligns or not with $\bar{y}$ [76]. Finally, we postulate the existence of an external authority (e.g. government policies, advertising campaign or mass media) promoting the new technology so that individuals get additional psychic benefit or pay psychic cost if they follow or not the recommendation of the external authority [111].

Electronic supplementary material, equation (S1) combines all these effects into a normative value *V*(*x*, *y*). Then the difference Δ*V*(*y*) = *V*(1, *y*) − *V*(0, *y*) in psychological pay-offs between choosing *x* = 1 and *x* = 0 for an individual with attitude *y* is

2.4 ΔV(y)=v(2y−1)⏟cognitive dissonance+k1(2p−1)⏟conformityw/peers′ actions+k2(2y¯−1)⏟conformityw/peers′ opinions+k3,⏟conformityw/authority

where parameters *v*, *k*_1_, *k*_2_ and *k*_3_ measure the effect of cognitive dissonance, of injunctive social norms, of conformity with peers and of conformity with (or trust to) the authority, respectively. All these parameters are individual-specific. Note that Δ*V*(*y*) depends of both the average behaviour (*p*) and the average attitude ($\bar{y}$) in the population.

### 2.3. The dynamics of attitudes

We postulate that after taking an action (i.e. choosing the technology to use) individuals observe the actions of their peers, get ideas about their average attitude through some interactions and are subject to influence by the external authority. As a result of these forces they then go through a psychological process of revising their attitude *y*. Specifically, we account for the effects of cognitive dissonance as well as for conformity with peers’ actions and attitudes towards the new technology, and with an external authority promoting its usage. Adapting the approach of Gavrilets [76], we describe these processes using a recurrence equation

2.5 y′−y=s[α(x−y)⏟cognitive dissonance+β1(p−y) ⏟conformityw/peers′ actions+β2(y¯−y)⏟conformityw/peers′ opinions+β3(1−y)⏟conformityw/authority].

The four terms in the brackets act to align the individual’s attitude *y* with their action *x*, with the average action *p* of their peers, with the average attitude $\bar{y}$ of peers and with the action *x* = 1 promoted by the authority, respectively. Parameter *s* measures the speed of change in attitude while parameters *α*, *β*_1_, *β*_2_ and *β*_3_ are the relative strengths of cognitive dissonance and the three conformity forces (*α* + *β*_1_ + *β*_2_ + *β*_3_ = 1). Parameter *β*_3_ also reflects the trust to the message of the authority. All these parameters are individual-specific. Parameters of the model are summarized in table 1.

**Table 1.** Most important variables, functions and parameters (also see electronic supplementary material, table S1).

	symbols	their meaning
variables	*x*	individual choice: old (*x* = 0) or new (*x* = 1) technology
	*y*	individual attitude towards the new technology (0 ≤ *y* ≤ 1)
	*b*	individual benefit/skill of using the new technology
functions	*p*	frequency of adopters, $p=\sum x_{i}/N$
	*a*(*p*)	individual learning rate, *a*(*p*) = *a*_0_ + *a*_1_ *p*
	Π(x,b)	perceived material benefit, equation (2.3)
	*V*(*x*, *y*)	normative component in the utility function, electronic supplementary material, equation (S1)
	*U*(*x*, *y*, *b*)	utility function, equation (2.2)
parameters	*b*_0_	individual benefit of using old technology
	*b*_min_, *b*_max_	minimum and maximum benefits of using new technology
	*c*	knowledge depreciation rate
	*a*_0_, *a*_1_	learning parameters
	*ω*	foresight parameter
	*v*, *α*, *D*	cognitive dissonance parameters (*D* = (*v* + *α*)/2)
	*k*_1_, *β*_1_, *K*_1_	conformity with peers’ actions parameters (*K*_1_ = (*k*_1_ + *β*_1_)/2)
	*k*_2_, *β*_2_, *K*_2_	conformity with peers’ attitudes parameters (*K*_2_ = (*k*_2_ + *β*_2_)/2)
	*k*_3_, *β*_3_, *K*_3_	conformity with authority parameters (*K*_3_ = (*k*_3_ + *β*_3_)/2)
	*ɛ*	strength of normative factors in the utility function
	*λ*	precision parameter
control variables	*p*_0_, *b*_*s*_	proportion of trained individuals and the subsidy to first adopters
	*f*_0_, *f*_1_, *f*_3_, *f*_4_	parameters scaling the effects of cognitive dissonance, visibility of peers’ actions, visibility of peers’ attitudes, and authority’s effort

Before proceeding with analysis, we want to stress two things. First, all socio-psychological factors included in our model have been repeatedly shown to be important in decision-making (see the references above). Second, the linear functions we used to capture these effects (see equations (2.4) and (2.5)) are both the simplest possible mathematically and also standard in models of social behaviour. For example, the terms analogous to components *v*(2*y* − 1), *k*_1_(2*p* − 1), $k_{2}(2\bar{y}-1)$ and *k*_3_ of the utility function present in equation (2.4) are present in various game-theoretic models (e.g. [51,76,112–115]). Moreover, the linear relationships can be very easily tested statistically on real data. Similarly, our equation (2.5) is an adaptation of the standard approach in social influence models [70–73,116–118] which use linear equations for describing the dynamics of personal attitudes and opinions as a result of the exchange of opinions between group members.

## 3. Results

We have analysed this model using numerical simulations and analytical approximations. We will start by discussing analytical results on equilibria when there is no variation in individual parameters and then follow with numerical studies of the general model. We will focus on the effects of various parameters on the frequency of adopters *p* and individual attitudes *y*. At the end, we will examine the effectiveness of different intervention strategies aiming to promote the diffusion of the new technology and how their effectiveness depends on cultural characteristics of the society.

### 3.1. Equilibria in the symmetric case

Assume that variation in individual parameters is absent and there are no errors in the decision-making process (*λ* = ∞). In this case, the system converges to an equilibrium where each adopter attains the maximum benefit of using the new technology *b*_max_ and each non-adopter has the minimum benefit of the new technology *b*_min_.

**Figure 2.**
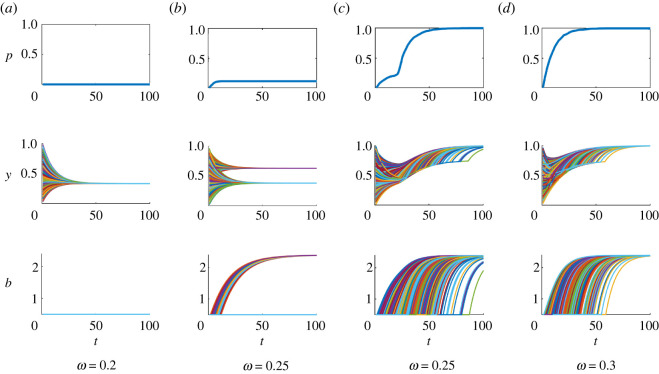
Convergence to equilibria in four independent runs with no between-individual variation and no errors in decision-making. Shown are: the frequency of adopters *p* (top row) individual attitudes *y* (middle row), and individual benefits *b* (bottom row). Different individuals are shown by different colours. The values of parameter *ω* are shown below the graphs. Other parameters: *b*_min_ = 0.5, *b*_0_ = 1, *b*_max_ = 2.4, *a*_0_ = *a*_1_ = 0.05, *c* = 0.1, *v* = *k*_1_ = *k*_2_ = *k*_2_ = *α* = *β*_1_ = *β*_2_ = *β*_3_ = 0.25, *ɛ* =0.5, *s* = 0.1, *N* = 1000, *ν* = 0.1, *λ* = ∞. Initial values of *y* are drawn randomly and independently from a beta distribution with mean 0.5 and standard deviation 0.2.

There are three possible types of equilibria: a complete failure of the new technology (so that p* = 0), a complete replacement of the old technology by the new one (so that p* = 1) and the coexistence of two technologies (so that 0 < p* < 1), respectively. The equilibria of the first two types will be called homogeneous, and the equilibria of the last type will be called heterogeneous. Figure 2 illustrates convergence to different equilibria.

At the equilibrium with p* = 0 (see figure 2*a*), where nobody uses the new technology, individuals still have some positive attitude towards it

$$y^{*}=\frac{\beta_{3}}{1-\beta_{2}},$$

due to the influence of the external authority. This attitude increases with *β*_3_ and the strength of conformity with peers’ attitude *β*_2_. The conditions for local stability of this equilibrium are given in the electronic supplementary material, SM. A sufficient condition for its instability and, thus, for some use of the new technology (i.e. for p* > 0) is

$$\Delta \Pi (b_{\min})+\epsilon k_{3}>\epsilon (v+k_{1}+k_{2}),$$

that is, the perceived material gain from using new technology for the first time ($\Delta \Pi (b_{\min})=\Pi (1,b_{\min})-\Pi (0,b_{\min})$) plus the effect of the external authority (ε*k*_3_) is larger than the joint effect of cognitive dissonance (ε*v*) and conformity with peers (ε*k*_1_, ε*k*_2_).

At equilibrium with p* = 1 (figure 2*c*,*d*), all individuals have the maximum attitude towards the new technology, i.e. y* = 1; this equilibrium is always locally stable under our assumptions about parameters.

The heterogeneous equilibria (figure 2*b*) form a ‘line’ of equilibria with *p*_low_ < p* < *p*_high_, where the boundaries *p*_low_, *p*_high_ are defined in the electronic supplementary material. At the heterogeneous equilibria, the attitudes of adopters and non-adopters differ by the value equal to the strength of cognitive dissonance: $y_{a}^{*}-y_{n}^{*}=\alpha$. If *β*_2_ = 0 (so that, there are no direct discussions between individuals), the expressions for $y_{a}^{*}$ and $y_{n}^{*}$ take a particularly simple form:

3.1 $$y_{a}^{*}=\alpha +(1-\alpha -\beta_{3})p^{*}+\beta_{3}\;\mathrm{and}\;y_{n}^{*}=(1-\alpha -\beta_{3})p^{*}+\beta_{3}.$$

As expected, both attitudes increase with the frequency of adopters (p*). The corresponding equations for the case of *β*_2_ > 0 are considered in the electronic supplementary material.

Interestingly, different heterogeneous equilibria can be simultaneously stable with other heterogeneous equilibria as well as with the homogeneous equilibria with p* = 0 and p* = 1, so that the eventual outcome can depend on initial conditions and chance. For example, figure 2*b*,*c* illustrates the coexistence of two types of equilibria: depending on the initial values of attitudes *y*, the system either converges to a heterogeneous equilibrium (shown in figure 2*b*), or to a homogeneous equilibrium with all individuals using the new technology (shown in figure 2*c*).

### 3.2. Agent-based simulations

To study more complex cases of our model where analytical progress is impossible we use agent-based simulations. In our simulations, individual parameters *v*, *k*_1_, *k*_2_ and *k*_3_ are chosen from a broken stick distribution on [0, 1]. Similarly, parameters *α*, *β*_1_, *β*_2_ and *β*_3_ are chosen from a broken stick distribution on [0, 1] as well. (The advantage of the broken-stick distribution [119,120] is that it has no parameters.) We also assume that individual values of parameters *a*_1_, *ω* and ε are drawn randomly and independently from truncated normal distributions with means ${\bar{a}}_{1},\bar{\omega },\bar{\varepsilon }$ and standard deviations *σ*_*a*1_, *σ*_*ω*_ and *σ*_ε_, respectively. Parameters *c* = 0.1, *b*_0_ = 1, *ν* = 0.1, *s* = 0.1, *N* = 1000, *λ* = 15 are constant. Unless otherwise stated, $a_{0}=0.05,\bar{a_{1}}=0.05$. Initial attitudes towards new technology are drawn randomly and independently from a beta distribution with a small mean value ${\bar{y}}_{0}$ (unless otherwise stated, ${\bar{y}}_{0}=0.1$) and standard deviation 0.2. We assume that initially the number of new technology adopters is zero (i.e. *x* = 0 for each individual). Initially, the benefit of new technology is minimal (i.e. *b* = *b*_min_).

With finite precision, the system converges asymptotically either to the equilibrium with p* = 0 or p* = 1. In general, the time to convergence can be large so that transient dynamics are of more relevance and interest. Therefore, we focus on the behaviour of the system at some time step *T*. In simulations, we consider three different time steps *T* = 50, 100 and 200. The choice of other parameters is discussed below. Unless otherwise stated, the results shown are based on 100 runs for each parameter combination.

#### 3.2.1. Effects of perceived material pay-offs

**Figure 3.**
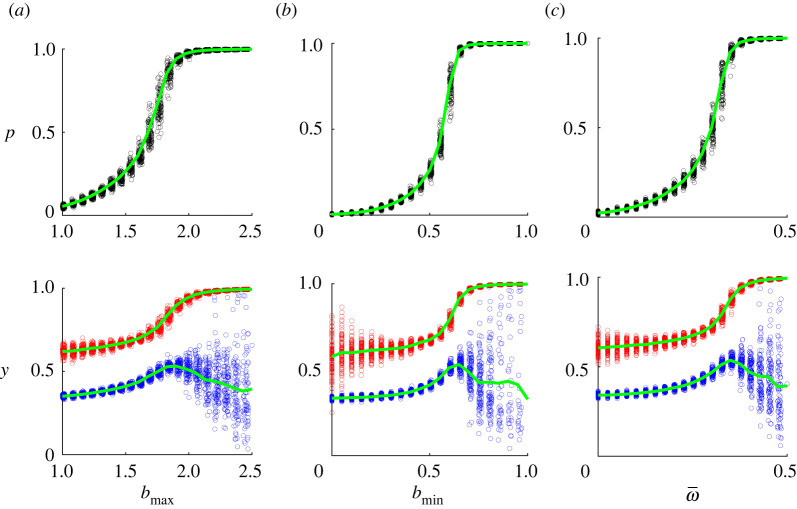
The dependence of the frequency of adopters *p* and attitudes *y* on: (*a*) the maximum benefit parameter *b*_max_, (*b*) the minimum benefit parameter *b*_min_, (*c*) the foresight parameter $\bar{\omega }$. Each point corresponds to an outcome of a particular run. Characteristics were calculated at *T* = 100. (The graphs corresponding to *T* = 50 and 200 are very similar to the ones shown.) Attitudes of adopters and non-adopters are marked in red and blue colours, respectively. Curves show the average values of corresponding characteristics among all runs. Baseline parameters: $b_{\min}=0.5,b_{\max}=1.5,{\bar{a}}_{1}=0.05,\bar{\omega }=0.25,\bar{\varepsilon }=0.5,\sigma_{a_{1}}=0.005,\sigma_{\omega }=0.025,\sigma_{\varepsilon }=0.05$.

Intuitively, increasing the maximum *b*_max_ and minimum *b*_min_ benefit of new technology as well as increasing the weight *ω* individuals put on future material benefits should simplify the spread of new technology. Results of numerical simulations support these intuitions. Figure 3 shows that the frequency of adopters as well as the attitudes of individuals increase with *b*_max_, *b*_min_ and $\bar{\omega }$. The difference between attitudes of adopters and non-adopters is approximately equal to the average of the cognitive dissonance parameter $\bar{\alpha }$ which is in line with our analytical results above. Note that the effects of parameters on frequency *p* are large relative to their effects on attitudes *y*. This happens because they have direct effects on individuals’ actions, while their effects on individual attitudes are indirect.

Figure 3 also shows some decline in the attitude of non-adopters for large values of *b*_max_, *b*_min_ and $\bar{\omega }$ when *p* becomes close to one. What happens is that a small number of remaining non-adopters represent a very biased sample of individuals characterized by very strong cognitive dissonance (i.e. large values of *v*_0_ and *α*) in comparison with adopters. Typically, non-adopters also have low sensitivity to the influence of the external authority (i.e. small *k*_3_ and *β*_3_) compared with those who adopt the new technology. All these differences are significant at the 10% level. For more details, see electronic supplementary material, figure S2. Also, there is a large variation in average attitudes of non-adopters between different runs if *p* is close to 1, that is, when there are few non-adopters. Their average attitude is then highly dependent on attitudes of each of these few individuals, so that it is highly dependent on initial attitudes and other model parameters (such as *a*_1_, *ω*, *ɛ*) which are randomly drawn from fixed distributions.

#### 3.2.2. Effects of policy interventions

Here we examine the effectiveness of five different intervention strategies aiming to promote the diffusion of a new technology in the society: training individuals, providing subsidies for early adopters, increasing the visibility of peer actions, simplifying the exchange of opinions between people and increasing the effort of the external authority to promote the innovation.

##### 3.2.2.1. Training individuals

**Figure 4.**
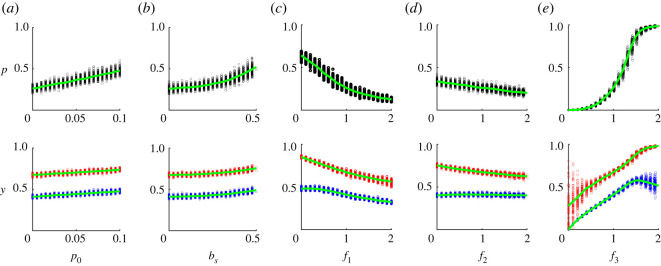
The dependence of the frequency of adopters *p* and attitudes *y* on: (*a*) the fraction of trained individuals *p*_0_, (*b*) the subsidy to early adopters *b*_*s*_, (*c*) increasing the visibility of peer actions *f*_1_, (*d*) simplifying the exchange of opinions between people *f*_2_, (*e*) increasing the effort of the external authority *f*_3_. Each point corresponds to an outcome of a particular run. Characteristics were calculated at *T* = 100. Attitudes of adopters and non-adopters are shown in red and blue colours, respectively. Curves show the average values of corresponding characteristics across all runs. Baseline parameters: $b_{\min}=0.5,b_{\max}=1.5,{\bar{a}}_{1}=0.05,\bar{\omega }=0.25,\bar{\varepsilon }=0.5,\sigma_{a_{1}}=0.005,\sigma_{\omega }=0.025,\sigma_{\varepsilon }=0.05$.

Assume that initially a small random fraction *p*_0_ of individuals are trained to use the new technology. In our model, the effect of training can be captured by assuming these individuals initially have maximum skills (*b* = *b*_max_), choose the new technology (i.e. their *x* is set to 1), and have the maximum attitude *y* = 1 towards it. Initial conditions for all other individuals are generated as specified above. As expected, increasing the frequency of trained individuals *p*_0_ simplifies the spread of new technology (figure 4*a*). As we show in the electronic supplementary material, figure S3, this effect intensifies with an increase in the strength of normative factors $\bar{\varepsilon }$. The reason is *p*_0_ affects individual actions and attitudes through cognitive dissonance and conformity with peers, which are stronger with larger *ɛ*.

##### 3.2.2.2. Material support for early adopters

One of the widely used strategies to influence the diffusion process is to support early adopters through subsidies or tax benefits for new technology users [121]. This can be captured in our model by assuming that each person who starts using the new technology in the first few time steps, receives an additional one-time subsidy in the amount of *b*_*s*_. Typically, introducing a subsidy *b*_*s*_ leads to an increase in *p* and *y* (figure 4*b*). We show in the electronic supplementary material, figure S4 that this strategy can be very efficient if the average foresight parameter $\bar{\omega }$ is relatively large, and the average effect of normative factors $\bar{\varepsilon }$ is not large. The outcome is intuitive: increasing *b*_*s*_ increases the perceived material pay-off associated with the new technology. Therefore, increasing *b*_*s*_ leads to a substantial increase in *p* if the perceived material pay-off associated with the new technology is relatively large and important in comparison with normative factors.

Next we investigate the efficiency of three additional strategies of promoting the spread of innovation: (i) increasing the visibility of peer actions, (ii) simplifying the exchange of opinions between people, and (iii) increasing the effort of the external authority. In the model, we introduce these strategies via additional factors *f*_*i*_ placed in front of the corresponding terms *k*_*i*_ and *β*_*i*_ of equations (2.4) and (2.5). For example, to study the effects of changing the effort of the external authority we would replace the terms *k*_3_ and *β*_3_(1 − *y*) in equations (2.4) and (2.5) with terms *f*_3_*k*_3_ and *f*_3_*β*_3_(1 − *y*). The changes in the effort of the external authority will then be modelled by changing the value of *f*_3_. While parameters *k*_1_, *k*_2_, *k*_3_ and *β*_1_, *β*_2_, *β*_3_ will vary between individuals, parameters *f*_1_, *f*_2_ and *f*_3_ will be the same for all individuals.

##### 3.2.2.3. Increasing the visibility of peer actions

A change of the visibility of peer actions can be modelled by changing the value of *f*_1_. Typically, increasing *f*_1_ decreases the frequency of adopters *p* and the attitudes *y* (figure 4*c*). The reason is that strong conformity with peers’ decisions makes it difficult to start using the new technology initially when no one uses it. However, as shown in electronic supplementary material, figure S5, increasing *f*_1_ can lead to a slight increase in *p* in some special cases of the model that are discussed in the electronic supplementary material (for more details see electronic supplementary material, §S3.4).

##### 3.2.2.4. Simplifying the exchange of opinions between people

This strategy involves creating a special environment in which individuals can discuss what they think about the new technology. Examples include creating special Internet forums or social events related to the new technology, where participants can meet each other and share their attitudes towards the new technology. In our model, this can be captured by increasing the factor *f*_2_. Typically, increasing *f*_2_ decreases the frequency of adopters *p* (figure 4*d*). The result is intuitive: initially all individuals have low attitudes towards the new technology. Therefore, increasing the strength of conformity with peers’ attitudes prevents the adoption in the early stages of the process. Increasing *f*_2_ coupled with a decay in *p* leads to a decrease in attitudes of adopters (through conformity with peers’ attitudes and actions, respectively). However, as shown in electronic supplementary material, figure S6, increasing *f*_2_ can lead to a slight increase in *p* in some special cases of the model that are discussed in the electronic supplementary material (for more details see electronic supplementary material, §S3.5).

##### 3.2.2.5. Increasing the effort of the external authority

Increasing the effort of the external authority to promote the new technology (e.g. through an advertising campaign or propaganda) is captured by increasing parameter *f*_3_. Figure 4*e* shows that increasing *f*_3_ leads to an increase in attitudes of all individuals (as long as *p* < 1). In general, the frequency of adopters *p* shows an S-shaped dependence on *f*_3_. This implies that relatively small increases in *f*_3_ can in some cases lead to a substantial increase in *p*. The effectiveness of this intervention strategy is higher for larger values of $\bar{\varepsilon }$ and larger $\bar{\omega }$ (see electronic supplementary material, figure S7). Figure 4*e* also shows some decline in the attitude of non-adopters for large values of *f*_3_. This is a general pattern observed if *p* is close to one. What happens is that a small number of remaining non-adopters represent a very biased sample of individuals characterized by very strong cognitive dissonance compared with adopters. Typically, non-adopters also have low sensitivity to the influence of their peers’ attitudes, and to the external authority compared with those who adopt the new technology. All these differences are significant at the 10% level. For more details, see electronic supplementary material, figure S8. We comment on the effects of the external authority further in the Discussion section.

#### 3.2.3. Effects of cognitive and psychological factors

As expected, increasing the minimum learning rate *a*_0_ can lead to a substantial increase in the frequency of adopters *p*. By contrast, the effect of parameter *a*_1_ characterizing the sensitivity of the learning rate *a* to the frequency of adopters is mostly insignificant. This is so because *a*_1_ has almost no effect on the learning rate in early stages when *p* ≈ 0, while the effect of *a*_0_ does not depend on *p* directly. Electronic supplementary material, figures S9 and S10 illustrate these conclusions.

Next we consider the effects of two additional parameters. One is the relative strength of normative factors ε in the utility function (2.2). (Recall that if *ɛ* = 0, individuals care only about perceived material pay-offs. On the other hand, if *ɛ* = 1, the weights of perceived material pay-offs and psychological factors in the utility function are the same.) The other factor is the relative strength of cognitive dissonance. To investigate it, we will introduce an additional parameter *f*_0_ placed in front of the terms *v*(2*y* − 1) and *α*(*x* − *y*) in equations (2.4) and (2.5), respectively, and varying its value. If *f*_0_ = 0, cognitive dissonance is absent. On the other hand if *f*_0_ = 1, its effects are comparable to those of conformity. Parameter *f*_0_ will be the same for all individuals in the population. It has been argued that some human cultures are more collectivist than others and the effects of cognitive dissonance in such cultures can be significantly weaker than those of conformity [122]. Similarly the effects of material pay-offs versus immaterial influences can also vary between cultures. Therefore, parameters $\bar{\varepsilon }$ and *f*_0_ can also be viewed as reflecting certain cultural aspects.

##### 3.2.3.1. Strength of normative factors *ɛ*

**Figure 5.**
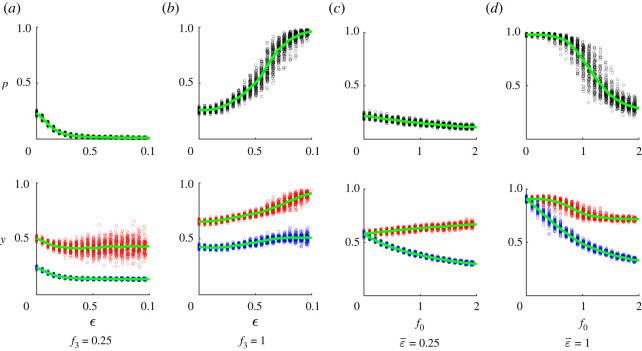
The dependence of the frequency of adopters *p* and attitudes *y* on: (*a*,*b*) the strength of normative factors *ɛ*, (*c*,*d*) the strength of cognitive dissonance *f*_0_. Each point corresponds to an outcome of a particular run. Characteristics were calculated at *T* = 100. Attitudes of adopters and non-adopters are shown in red and blue colours, respectively. Curves show the average values of corresponding characteristics across all runs. Baseline parameters: $b_{\min}=0.5,b_{\max}=1.5,{\bar{a}}_{1}=0.05,\sigma_{a_{1}}=0.005,\sigma_{\omega }=0.025,\sigma_{\varepsilon }=0.05$, (*a*,*b*) $f_{0}=1,\bar{\omega }=0.3$ and (*c*,*d*) $f_{3}=1,\bar{\omega }=0.25$.

Effects of the relative strength of normative factors $\bar{\varepsilon }$ depend on the strength of conformity with the authority controlled by parameter *f*_3_. For relatively small *f*_3_, increasing $\bar{\varepsilon }$ leads to a decrease in the frequency of adopters *p* coupled with a decrease in attitudes of all individuals (figure 5*a*). What happens is that with small *f*_3_, conformity with peers, who initially do not use the new technology, dominates so that the overall effect of normative factors is negative for almost all individuals. Consequently, increasing $\bar{\varepsilon }$ basically amplifies this negative effect, preventing the spread of the new technology.

Conversely, for relatively large *f*_3_, increasing the strength of normative factors $\bar{\varepsilon }$ increases the frequency of adopters *p* and attitudes *y* (figure 5*b*). What happens is that with large *f*_3_, conformity with the authority is strong so that the overall effect of normative factors is positive for a relatively large fraction of individuals. Increasing *ɛ* amplifies this positive effect. Electronic supplementary material, figure S11 provides further details on the interactions of different factors.

##### 3.2.3.2. Cognitive dissonance

Increasing the strength of cognitive dissonance, which is controlled by parameter *f*_0_ decreases the frequency of adopters *p* (figure 5*c*,*d*). The reason is that strong cognitive dissonance makes it difficult to start using a new technology initially when the attitude towards it is low. This effect is quite more marked if $\bar{\varepsilon }$ is large. Increasing *f*_0_ has two main effects on *y*: (i) it forces individuals to align their attitudes with actions and (ii) it affects individual actions, which, in turn, have an impact on *y* through the conformity term. The former effect is positive for adopters, and negative for non-adopters. Since increasing *f*_0_ decreases *p*, the latter effect is negative. As a result, increasing *f*_0_ decreases attitudes of non-adopters (figures 5*c*,*d*). Attitudes of adopters can either increase (figure 5*c*) or decrease (figure 5*d*) with an increase in *f*_0_, depending on the size of the above opposing effects. The difference between attitudes of adopters and non-adopters is amplified by increasing *f*_0_. For more details, see electronic supplementary material, figure S12.

#### 3.2.4. Dynamics of adoption

**Figure 6.**
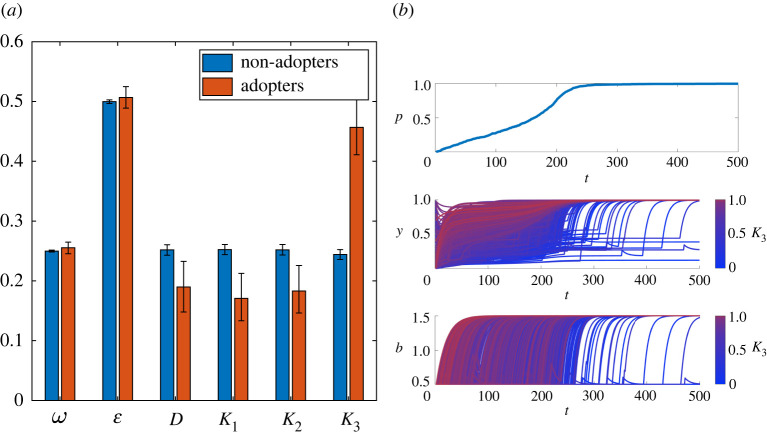
Dynamics of adoption. (*a*) Average characteristics of adopters and non-adopters at *T* = 10. The averages and the 95% confidence intervals are calculated among 1000 independent runs. (*b*) An example of a single run: the frequency of adopters *p*, individuals attitudes *y* and individuals benefits *b*. Different individuals are shown by different colours related to their value of parameter *K*_3_. Individuals with the highest values of parameter *K*_3_ measuring conformity with the authority are shown in red, while those with smallest *K*_3_ are shown in blue. Other parameters: $b_{\min}=0.5,b_{\max}=1.5,\bar{\omega }=0.25,\bar{\varepsilon }=0.5,\sigma_{a_{1}}=0.005,\sigma_{\omega }=0.025,\sigma_{\varepsilon }=0.05$.

Here we consider who become early adopters of the new technology in our model. To simplify notation, we introduce the overall strength of cognitive dissonance *D* = (*v* + *α*)/2, of conformity with peers’ action *K*_1_ = (*k*_1_ + *β*_1_)/2, with peers’ attitudes, *K*_2_ = (*k*_2_ + *β*_2_)/2, and with external authority *K*_3_ = (*k*_3_ + *β*_3_)/2. Figure 6*a* shows that early adopters are characterized by lower degrees of cognitive dissonance *D* and conformity with peers *K*_1_, *K*_2_ and significantly higher sensitivity *K*_3_ to the external influence. There is no difference between adopters and non-adopters in the foresight parameter *ω* and the overall strength of normative factor *ɛ*. All the above conclusions are drawn at the 5% significance level. Strong sensitivity to the message of the external authority is necessary to overcome the expected drop in material pay-offs as well as the effects of cognitive dissonance and conformity with peers. After early adopters start using the new technology, other individuals one-by-one switch to using it (figure 6*b*). These observations are in line with our discussion above.

## 4. Discussion

Social influences have been a crucial factor in human evolution and behaviour since the origin of our species [123,124]. Social influences are also very important in the success or failure of many technological innovations, which is a well-known fact also reflected in many mathematical models starting with [21]. Understanding and predicting group behaviours, including those related to the adoption of innovations, is impossible without accounting for the differences between individuals in how they react to social influences. Starting with the pioneer work of [125–129] there are now many theoretical studies explicitly dealing with such differences, including those describing innovation diffusion [22,83]. One important aspect of between-individual variation is the difference in their psychology, attitudes and beliefs, which can change in time as individuals learn to take advantage of the new technology and as various social interactions they are engaged in unfold. Here we have aimed to understand better the effects of these processes on innovation diffusion.

Using recent advances in modelling the dynamics of actions, attitudes and beliefs in social dilemmas [48,76], we have built a novel model describing co-evolution of attitudes of individuals towards the new technology, their adoption decisions and their abilities to take advantage of it. In our model, individual decisions and changes in attitudes are controlled by cognitive dissonance, conformity with observed peers’ decisions, conformity with peers’ attitudes (revealed in direct discussions, online, etc.), and by an external influence (e.g. advertising or mass media). Our focus was on manufacturing a product using a new technology which requires certain time investment to achieve a desired efficiency. However, our model also applies to consumers trying to take advantage of a new product.

**Figure 7.**
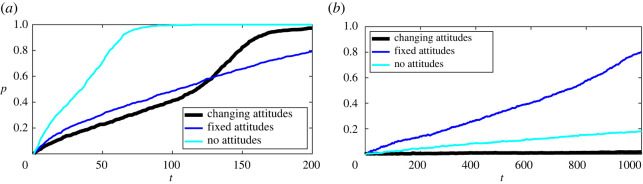
Effect of attitudes on the adoption curves. Examples of a single run of the model with dynamically changing attitudes (the black curve), fixed attitudes (the blue line, *s* = 0) and the model that does not take into account attitudes (the cyan line, *s* = 0 and *v* = *k*_2_ = *α* = *β*_2_ = 0 for all individuals) are shown. Baseline parameters: $b_{\min}=0.5,\sigma_{a_{1}}=0.005,\sigma_{\omega }=0.025,\sigma_{\varepsilon }=0.05$, (*a*) $b_{\max}=1.5,\bar{\omega }=0.25,\bar{\varepsilon }=0.5,f_{3}=1$ and (*b*) $b_{\max}=1.45,\bar{\omega }=0.3,\bar{\varepsilon }=0.45,f_{3}=0$.

There are many, largely overlapping, theories of behaviour and behavioural change across the social and behavioural sciences [130]. Our approach can be viewed as an extension of earlier continuous opinions and discrete actions (CODA) models [28,131,132] in which actions of individuals depend on their attitudes (opinions) which in turn depend on observed behaviour of others. Our approach is also related to social psychology approaches [22,133,134], where adoption decisions are based on psychological rules rather than perfect rationality. Specifically, our model can be viewed as an extension of models in [135,136] based on the theory of planned behaviour [137] which accounts for individual attitudes towards a technology and individual subjective norms. Earlier mathematical models have usually neglected the dynamic nature of individual attitudes. This, however, can lead to overestimation or underestimation of the number of adopters, as well as incorrect adoption curves (figure 7*a*). Moreover, ignoring the dynamic nature of attitudes can lead to wrong predictions of long-term equilibria (figure 7*b*). As a result, the predictive power of models and the effectiveness of the different policies based on them can be reduced.

While there is an extensive literature on contagion of products, services, ideas and technologies based on social influence [32,33], the main focus of this literature is on network aspects of the diffusion process. On the contrary, in our work we emphasize on the individual decision-making, the co-evolution of individual actions and attitudes, on psychological, social and cultural aspects of these processes. This allows us to look from a new angle at well-known intervention strategies frequently used by different policy-makers, as well as to make theoretically grounded predictions about the success or failure of a particular technology in different cultural, regional or religious contexts.

### 4.1. Intervention strategies

We have used our model to evaluate the effectiveness of five different intervention strategies aiming to promote the diffusion of a new technology: (i) training individuals, (ii) providing subsidies for early adopters, (iii) increasing the visibility of peer actions, (iv) simplifying the exchange of opinions between people, and (v) increasing the effort of an external authority (such as cultural, social or political leaders or commercial advertisements). Our results show that training and subsidies can help spreading the innovation. Although this conclusion is intuitive, our models allow one to evaluate the resulting effects quantitatively. Increasing the visibility of peer actions and simplifying the exchange of opinions between people can have negative effect. The underlying cause of this is conformity with peers which acts against trying something new when the great majority of peers still use the old technology. An important caveat of our conclusions is that we assumed that both training and between-peer interactions were completely random. Targeting individuals with particular attitudes or increasing visibility of early adopters are expected to increase the positive effects of these interventions.

### 4.2. Conformity with authority

Our results show that the effort of external influence (measured by parameter *f*_3_ in our model) is the most important factor in the initial spread of innovations. This is hardly surprising and is well supported by empirical studies. For example, government effort was found to be critical in encouraging the adoption of sustainable technology in the Malaysian SMEs sector [138]. Effects of an external influence (via advertising or mass media) have already been studied extensively [22,139–142]. It has been argued that advertising mostly contributes to the spread of initial awareness about the innovation, rather than to its adoption [22]. Goldenberg *et al.* [141] concluded that the effect of external influences is strong at early stages of the diffusion process and decreases in time. Here we assumed that all individuals were already aware of the new technology and the effort of the external authority was directed towards exploiting individual tendencies to comply with propaganda [143,144] by modifying both the behaviour and attitudes. By the authority we mean companies producing the innovation, governments, local or global authorities aiming to promote some strategic innovations, and/or opinion leaders or influential others agitating for a new product or technology. Our results show (figure 6) that it is the individuals who are most affected by the external influence who are the first to start using the new technology.

**Figure 8.**
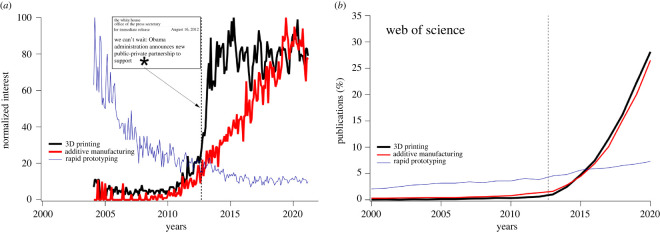
Effects of the President Obama’s announcement promoting the deployment of additive manufacturing. (*a*) Comparison of the normalized interest measured by Google Trends with reference to the technologies referred to as ‘rapid prototyping,’ ‘additive manufacturing,’ and '3D printing'. The normalized interest is measured as a search interest relative to the highest point on the chart for the given region and time. A value of 100 is the peak popularity for the term. A value of 50 means that the term is half as popular. A score of 0 means there was not enough data for this term. The vertical line corresponds to the month and year when President Obama announced the launch of the manufacturing institute mainly focused on additive manufacturing. (*b*) Comparison of normalized publications per year with key words additive manufacturing, and 3D printing shows rapid expansion after the launch date of the institute on additive manufacturing and overtaking the slowly evolving rapid prototyping technology. This is attributed to the slow realization of the benefits of this new technology by the engineering communities. The Web of Science data: the normalized publications per year is measured as the percentage of papers published that year that mention the corresponding term (3D printing, additive manufacturing, or rapid prototyping, respectively).

In our model, the effect of the external authority was limited to messaging about potential benefits of the new technology. However the authority can take a much more active and powerful role. For example, the adoption of the 3D printing (or additive manufacturing) technology was very slow during the 2000s, despite its demonstrated feasibility [145] due to engineering norms existing at that time [146]. (The terms ‘3D printing’ and ‘additive manufacturing’ refer to a technology that allows the engineer to realize a geometry conceived in a computer-aided design software to a product in one step by fusing various material feedstocks including powder, wire and tapes in a layer-by-layer fashion [147]. The foundational knowledge of this technology can be traced back to rapid prototyping, i.e. a process for rapidly creating the shape of a manufactured component for quick evaluation before releasing to mass production by traditional manufacturing processes that rely on pre-existing worldwide supply chain.) To accelerate the adoption of the 3D printing, leaders in the US government, academia and industry developed a focused effort to leverage emerging technologies in advanced manufacturing. As a part of this initiative, President Obama announced in June 2011 the launch of the public-private partnership dedicated to deployment of additive manufacturing [148] which was followed by additional initiatives in 2012 [149]. Figure 8 illustrates the dramatic effect of President Obama’s announcement on the adoption of additive manufacturing and the 3D printing as tracked by Google Trends and Web of Science data (see also [150]).

### 4.3. Psychological factors

We have also examined the effect of different psychological factors on the model dynamics. Our results show that individuals subject to strong cognitive dissonance as well as those exhibiting most conformity with the behaviour and attitudes of peers are less likely to be among the first adopters. On the other hand, simplifying learning process of the new technology increases its speed of adoption, as expected.

### 4.4. Effects of culture

Research shows that different geographic groups, cultures and countries can show significant differences in the psychological and normative characteristics of their populations. First, psychological research shows that cognitive dissonance is more likely to be expressed in some groups of individuals, societies and cultures than in others [122,151–153]. Second, societies and cultures vary in their ‘perception, preference, and social norms regarding time’ [154, p. 1]. Specifically, societies and cultures vary in their perception of the importance of future benefits and pay-offs [155]. In economic literature such time preferences are captured by a discounting rate. Cross-cultural work highlights the importance of culture in time discounting. Examples include studies of Canadian undergraduates and foreign undergraduates of Chinese descents [156], American, Chinese and Japanese graduate students living in the USA. [157], Israeli Arabs and Israeli Jews [158], as well as a comparison of 53 countries [159]. In psychology and social sciences, time preferences are reflected in a well-established cultural dimension of ‘future orientation’ which refers to the valuation of long-term versus short-term benefits [160,161]. Third, societies can vary in the extent to which individuals are influenced by actions and attitudes of others. For example, in collectivist societies individuals tend to align their actions with actions of their peers, while people in individualist societies care more about their own benefits and values [31,48,162].

In our model, the extent of future orientation (or the discounting rate) of a cultural group is controlled by the average value of the foresight parameter $\bar{\omega }$, the overall strength of normative factors relative to material pay-offs by the average value $\bar{\varepsilon }$, and the strength of cognitive dissonance by parameter *f*_0_. We initially introduced parameters *f*_1_, *f*_2_ and *f*_3_ as measures of the strength of three different policy interventions. However, they can also be interpreted as culture-related measures of different types of the conformity in the society. For example, societies with small *f*_1_ and *f*_2_ can be viewed as individualist, while those with large *f*_1_ and *f*_2_ as collectivist; and societies with large *f*_3_ can be interpreted as those with a strong norm of conformity with (and trust to) the authority.

Looking at our results from this angle allows us to conclude that cultures most prone to adapting innovations will be more future-oriented and more sensitive to the message of the authority promoting the new technology. Existing data show that indeed future-oriented cultures encourage individuals to adopt a new technology, which can increase long-term economic performance [163], while (as discussed above) societies with strong conformity with the authority can exhibit high rates of innovations, if, for example, the authority prioritizes innovative activities [164].

Our model also predicts that societies with stronger cognitive dissonance are more resistant to new technologies and exhibit a higher difference between attitudes of adopters and non-adopters. In real life, adopters (or non-adopters) can organize in a community through, for example, Internet forums, which will intensify communications between the members of the community and prevent communications with individuals outside the group (because they have significantly different attitudes). As a result, the society may contain a number of groups of individuals using different technologies.

Our model predicts that, typically, individualist societies are more successful in the diffusion of new technologies than collectivist societies, especially in early stages of adoption. This result is well in line with the recent conclusions of [165], which showed that individualism has a positive effect on the diffusion speed in the early stage of adoption. We have also found that collectivist culture can be more successful in later stages of adoption in societies with high future orientation and strong sensitivity and trust to the message of the authority. This arises because future orientation and conformity with the authority mitigate negative effects of conformity with peers in early stages of adoption.

Finally, our results imply that societies with strong normative factors can be very successful in adopting the new technology if they have a strong norm of conformity with the authority. Conversely, if conformity with peers and/or cognitive dissonance dominate in individual decision-making, societies with a larger strength of normative factors are less successful. We have to stress though that our model does not include many other important factors affecting the spread of innovations such as economic, political and social influences and circumstances.

### 4.5. Tight–loose cultures and innovations

Empirical studies show that different countries, cultures and groups can vary in tightness–looseness scale. Tight societies are characterized by strong norms (e.g. personal, social or norms supported by governments or other authorities), and low tolerance to deviant behaviours, while loose societies are the opposite [166–168]. In our model, the strength of norms can be captured by parameter *ɛ* so that loose societies have lower values of *ɛ* than tight ones. Moreover, as pointed out in [169], loose societies are more diverse in terms of opinions and attitudes, because their culture encourages deviations and tolerates mistakes. As a result, we assume that the levels of heterogeneity in our model parameters and in initial attitudes towards the new technology are higher in loose societies, which promotes the spread of the new technology.

However, as shown in empirical research, some tight societies can be very successful in terms of innovativeness. For example, Chinese provinces with tighter cultures have higher rates of incremental innovations than looser provinces [164]. This can also be explained by the fact that the Chinese government has prioritized innovations over the past decades, which, coupled with a strong norm of conformity with the authority, fosters innovation diffusion in tight provinces. This empirical observation is well in line with our theoretical results: increasing the strength of normative factors *ɛ* can promote the strength of a new technology if there is a strong norm of conformity with the authority supporting this technology.

### 4.6. Social norms

Our results are also relevant for research on social norms [44–47,74,100,102,170–172]. In particular, variable *y* can be viewed as a personal norm with individuals experiencing dis-utility if their action *x* deviates from *y*. Individuals also experience dis-utility if their action deviates from the average behaviour and the average attitude in the group which can be viewed as measures of the descriptive and injunctive norms in the population. The loss of utility due to the deviation from the behaviour promoted by an external authority can also be viewed as emerging due to the injunctive norm imposed on the group. A novel component of our model relative to earlier modelling work on social norms [48,74,98,99,102] is that we allow for the personal value/attitude *y* to change. We have also accounted for additional factors such as different types of conformity.

### 4.7. Limitations and possible generalizations

Several limitations of our study should be pointed out. First, our model does not take into account the structure of interactions within the population assuming that all individuals have exactly the same information about their peers. The model can be generalized by assuming that interactions between individuals happen on a network [26–29]. This would allow us to study the effect of interactions between different socio-psychological and topological factors on the diffusion process. This will also allow us to model explicitly the effects of opinion leaders [141,173–176] and to examine the effectiveness of different more comprehensive targeting intervention strategies, e.g. targeting opinion leaders, different small groups of individuals located in different places of a network, or a small number of large groups [177]. One can also elaborate the model by adding a hierarchical group structure. With this extension, one can recognize different social norms, e.g. conformity with group-mates or global conformity. This would allow one to study co-evolution of diffusion processes in different ethnic or religious sub-populations within a society [178]. We have assumed that individuals make their decisions maximizing the utility function (with random errors). However, other strategy revision protocols can be considered, like simple repetition [133], reinforcement learning [179] or selective imitation [103]. We leave these extensions and generalizations for future work.

In our model, we did not consider other potential factors promoting the initial spread of innovations like status seeking or novelty seeking [180,181]. Neither did we analyse an additional potentially efficient strategy which is manipulating people’s beliefs about the efficiency of the innovation and about the frequency of people who have already adapted it.

## Data Availability

The simulations were performed in MATLAB (Version R2019b). Data and relevant code for this research work are stored in GitHub: https://github.com/dtverskoi/The-spread-of-technological-innovations-effects-of-psychology-culture-and-policy-interventions and have been archived within the Zenodo repository: https://doi.org/10.5281/zenodo.5715314 [182]. Electronic supplementary material is available online [183].

## References

[1] Abramovitz M. 1956 Resource and output trends in the United States since 1870. Am. Econ. Rev. **46**, 5-23.

[2] Solow R. 1957 Technical change and the aggregate production function. Rev. Econ. Stat. **39**, 312-320. (10.2307/1926047)

[3] Currie T, Turchin P, Turner E, Gavrilets S. 2020 Duration of agriculture and distance from the steppe predict the evolution of large-scale human societies in Afro-Eurasia. Humanit. Soc. Sci. Commun. **7**, 34. (10.1057/s41599-020-0516-2)

[4] Turchin P, Currie T, Turner EAL, Gavrilets S. 2013 War, space, and the evolution of Old World complex societies. Proc. Natl Acad. Sci. USA **109**, 14 069-14 074. (10.1073/pnas.1201718109) PMC3799307 24062433

[5] Gong G, Keller W. 2003 Convergence and polarization in global income levels: a review of recent results on the role of international technology diffusion. Res. Policy **32**, 1055-1079. (10.1016/S0048-7333(02)00136-1)

[6] Parente S, Prescott E. 1994 Barriers to technology adoption and development. J. Pol. Econ. **102**, 298-321. (10.1086/261933)

[7] Comin D, Hobijn B. 2011 Technology diffusion and postwar growth. NBER Macroecon. Ann. **25**, 209-246. (10.1086/657531)

[8] Baptista R. 1999 The diffusion of process innovations: a selective review. Int. J. Econ. Bus. **6**, 107-129. (10.1080/13571519984359)

[9] Schumpeter J. 1934 The theory of economic development. Cambridge, MA: Harvard University Press.

[10] Peres R, Muller E, Mahajan V. 2010 Innovation diffusion and new product growth models: a critical review and research directions. Int. J. Res. Mark. **27**, 91-106. (10.1016/j.ijresmar.2009.12.012)

[11] Saaksjarvi M. 2003 Consumer adoption of technological innovations. Eur. J. Innov. Manage. **6**, 90-100. (10.1108/14601060310475246)

[12] Deffuant G, Huet S, Amblard F. 2005 An individual-based model of innovation diffusion mixing social value and individual benefit. AJS **110**, 1041-1069. (10.1086/430220)

[13] Young H. 2006 The diffusion of innovations in social networks. In The economy as an evolving complex system III: current perspectives and future directions (eds LE Blume, SN Durlauf). Oxford Scholarship Online. (10.1093/acprof:oso/9780195162592.001.0001)

[14] Liebermann Y, Paroush J. 1982 Economic aspects of diffusion models. J. Econ. Bus. **34**, 95-100. (10.1016/0148-6195(82)90021-2)

[15] Robertson T, Gatignon H. 1986 Competitive effects on technology diffusion. J. Mark. **50**, 1-12. (10.1177/002224298605000301)

[16] Acemoglu D, Robinson J. 2000 Political losers as a barrier to economic development. Am. Econ. Rev. **90**, 126-130. (10.1257/aer.90.2.126)

[17] Comin D, Hobijn B. 2009 Lobbies and technology diffusion. Rev. Econ. Stat. **91**, 229-244. (10.1162/rest.91.2.229)

[18] Milner H. 2006 The digital divide: the role of political institutions in technology diffusion. Comp. Pol. Stud. **39**, 176-199. (10.1177/0010414005282983)

[19] Chien-fei C, Xu X, Arpan L. 2017 Between the technology acceptance model and sustainable energy technology acceptance model: investigating smart meter acceptance in the United States. Energy Res. Soc. Sci. **25**, 93-104. (10.1016/j.erss.2016.12.011)

[20] Godin B. 2014 Invention, diffusion and linear models of innovation: the contribution of anthropology to a conceptual framework. J. Innov. Econ. Manage. **3**, 11-37. (10.3917/jie.015.0011)

[21] Bass F. 1969 A new product growth for model consumer durables. Manage. Sci. **15**, 215-227. (10.1287/mnsc.15.5.215)

[22] Kiesling E, Günther M, Stummer C, Wakolbinger L. 2012 Agent-based simulation of innovation diffusion: a review. Cent. Eur. J. Oper. Res. **20**, 183-230. (10.1007/s10100-011-0210-y)

[23] Mahajan V, Muller E. 1979 Innovation diffusion and new product growth models in marketing. J. Mark. **43**, 55-68. (10.1177/002224297904300407)

[24] Meade N, Islam T. 2006 Modelling and forecasting the diffusion of innovation – a 25-year review. Int. J. Forecast. **22**, 519-545. (10.1016/j.ijforecast.2006.01.005)

[25] Chatterjee R, Eliashberg J. 1990 The innovation diffusion process in a heterogeneous population: a micromodeling approach. Manage. Sci. **36**, 1057-1079. (10.1287/mnsc.36.9.1057)

[26] Bala V, Goyal S. 1998 Learning from neighbours. Rev. Econ. Stud. **65**, 595-621. (10.1111/1467-937X.00059)

[27] Kreindler G, Young H. 2014 Rapid innovation diffusion in social networks. Proc. Natl Acad. Sci. USA **111**, 10 881-10 888. (10.1073/pnas.1400842111) PMC4113916 25024191

[28] Martins A, Pereira C, Vicente R. 2009 An opinion dynamics model for the diffusion of innovations. Physica. A **388**, 3225-3232. (10.1016/j.physa.2009.04.007)

[29] Montanari A, Saberi A. 2010 The spread of innovations in social networks. Proc. Natl Acad. Sci. USA **107**, 20 196-20 201. (10.1073/pnas.1004098107) PMC2996710 21076030

[30] Goldenberg J, Han S, Lehmann D, Hong J. 2009 The role of hubs in the adoption process. J. Mark. **73**, 1-13. (10.1509/jmkg.73.2.1)

[31] Desmarchelier B, Fang E. 2016 National culture and innovation diffusion: exploratory insights from agent-based modeling. Technol. Forecast. Soc. Change **105**, 121-128. (10.1016/j.techfore.2016.01.018)

[32] Aral S, Walker D. 2011 Creating social contagion through viral product design: a randomized trial of peer influence in networks. Manage. Sci. **57**, 1623-1639. (10.1287/mnsc.1110.1421)

[33] Iyengar R, Van den Bulte C, Valente TW. 2011 Opinion leadership and social contagion in new product diffusion. Mark. Sci. **30**, 195-212. (10.1287/mksc.1100.0566)

[34] Bhojvaid V, Jeuland M, Kar A, Lewis JJ, Pattanayak SK, Ramanathan N, Ramanathan V, Rehman IH. 2014 How do people in rural India perceive improved stoves and clean fuel? Evidence from Uttar Pradesh and Uttarakhand. Int. J. Environ. Res. Public Health **11**, 1341-1358. (10.3390/ijerph110201341) 24473110 PMC3945541

[35] Agarwal B. 1983 Diffusion of rural innovations: some analytical issues and the case of wood-burning stoves. World Dev. **11**, 359-376. (10.1016/0305-750X(83)90047-5)

[36] Misra K et al. 1975 Safe water in rural areas. An experiment in promoting community participation in India. Int. J. Health Educ. **18**, 53-59.

[37] Park S. 2004 Quantitative analysis of network externalities in competing technologies: the VCR case. Rev. Econ. Stat. **86**, 937-945. (10.1162/0034653043125275)

[38] Merton RK. 1948 The self fulfilling prophecy. Antioch Rev. **8**, 173-190. (10.2307/4609267)

[39] Barzilai-Nahon K, Barzilai G. 2005 Cultured technology: the internet and religious fundamentalism. Inf. Soc. **21**, 25-40. (10.1080/01972240590895892)

[40] Kleijnen M, Wetzels M, De Ruyter K. 2004 Consumer acceptance of wireless finance. J. Financ. Ser. Mark. **8**, 206-217. (10.1057/palgrave.fsm.4770120)

[41] Loch K, Straub D, Kamel S. 2003 Diffusing the Internet in the Arab world: the role of social norms and technological culturation. IEEE Trans. Eng. Manage. **50**, 45-63. (10.1109/TEM.2002.808257)

[42] Sawang S, Sun Y, Salim S. 2014 It’s not only what I think but what they think! The moderating effect of social norms. Comput. Educ. **76**, 182-189. (10.1016/j.compedu.2014.03.017)

[43] Webster J, Trevino L. 1995 Rational and social theories as complementary explanations of communication media choices: two policy-capturing studies. Acad. Manage. J. **38**, 1544-1572. (10.5465/256843)

[44] Bicchieri C. 2006 The grammar of society: the nature and dynamics of social norms. Cambridge, UK: Cambridge University Press.

[45] Cialdini R, Reno R, Kallgren C. 1990 A focus theory of normative conduct: recycling the concept of norms to reduce littering in public places. J. Pers. Soc. Psychol. **58**, 1015-1026. (10.1037/0022-3514.58.6.1015)

[46] Kallgren C, Reno R, Cialdini R. 2000 A focus theory of normative conduct: when norms do and do not affect behavior. Pers. Soc. Psychol. Bull. **26**, 1002-1012. (10.1177/01461672002610009)

[47] Reno R, Cialdini R, Kallgren C. 1993 The transsituational influence of social norms. J. Pers. Soc. Psychol. **64**, 104-112. (10.1037/0022-3514.64.1.104)

[48] Gavrilets S. 2020 The dynamics of injunctive social norms. Evol. Hum. Sci. **2**, e60. (10.1017/ehs.2020.58) PMC10427483 37588350

[49] Ushchev P, Zenou Y. 2017 Technology adoption and social norms. See https://scholar.google.com/scholar?hl=ru&as_sdt=0%2C43&q=Ushchev%2C+P.+and+Zenou%2C+Y.+%282017%29.+Technology+adoption+and+social+norms&btnG=.

[50] Young H. 2011 The dynamics of social innovations. Proc. Natl Acad. Sci. USA **108**, 21 285-21 291. (10.1073/pnas.1100973108) PMC3271568 22198762

[51] Le Coent P, Preget R, Thoyer S. 2018 Do farmers follow the herd? The influence of social norms in the participation to agri-environmental schemes. HAL Open Science. See https://halshs.archives-ouvertes.fr/halshs-01936004/document.

[52] Festinger L. 1962 Cognitive dissonance. Sci. Am. **207**, 93-106. (10.1038/scientificamerican1062-93) 13892642

[53] McGrath A. 2017 Dealing with dissonance: a review of cognitive dissonance reduction. Soc. Personal Psychol. Compass **11**, e12362. (10.1111/spc3.12362)

[54] Marikyan D, Papagiannidis S, Alamanos E. 2020 Cognitive dissonance in technology adoption: a study of smart home users. Inf. Syst. Front., 1-23. (10.1007/s10796-020-10042-3) 32837263 PMC7381864

[55] Park I, Cho J, Rao H. 2015 The dynamics of pre-and post-purchase service and consumer evaluation of online retailers: a comparative analysis of dissonance and disconfirmation models. Decis. Sci. **46**, 1109-1140. (10.1111/deci.12176)

[56] Venkatesh V, Goyal S. 2010 Expectation disconfirmation and technology adoption: polynomial modeling and response surface analysis. MIS Q. **34**, 281-303. (10.2307/20721428)

[57] Briley DA, Morris MW, Simonson I. 2000 Reasons as carriers of culture: dynamic versus dispositional models of cultural influence on decision making. J. Consum. Res. **27**, 157-178. (10.1086/314318)

[58] Chang LJ, Sanfey AG. 2013 Great expectations: neural computations underlying the use of social norms in decision-making. Soc. Cogn. Affect. Neurosci. **8**, 277-284. (10.1093/scan/nsr094) 22198968 PMC3594719

[59] Guess CD. 2004 Decision making in individualistic and collectivistic cultures. Online Read. Psychol. Cult. **4**, 1-18.

[60] Harmon-Jones E, Harmon-Jones C. 2002 Testing the action-based model of cognitive dissonance: the effect of action orientation on postdecisional attitudes. Pers. Soc. Psychol. Bull. **28**, 711-723. (10.1177/0146167202289001)

[61] Jarcho JM, Berkman ET, Lieberman MD. 2011 The neural basis of rationalization: cognitive dissonance reduction during decision-making. Soc. Cogn. Affect. Neurosci. **6**, 460-467. (10.1093/scan/nsq054) 20621961 PMC3150852

[62] Melnyk V, van Herpen E, Trijp H. 2010 The influence of social norms in consumer decision making: a meta-analysis. ACR North Am. Adv. **37**, 463-464. (10.1027/2151-2604/a000352)

[63] Sanfey AG, Stallen M, Chang LJ. 2014 Norms and expectations in social decision-making. Trends Cogn. Sci. **18**, 172-174. (10.1016/j.tics.2014.01.011) 24582437

[64] Calabuig V, Olcina G, Panebianco F. 2017 The dynamics of personal norms and the determinants of cultural homogeneity. Ration. Soc. **29**, 322-354. (10.1177/1043463117717233)

[65] Calabuig V, Olcina G, Panebianco F. 2018 Culture and team production. J. Econ. Behav. Org. **149**, 32-45. (10.1016/j.jebo.2018.03.004)

[66] Kuran T. 1989 Sparks and prairie fires: a theory of unanticipated political revolution. Public Choice **61**, 41-74. (10.1007/BF00116762)

[67] Kuran T, Sandholm WH. 2008 Cultural integration and its discontents. Rev. Econ. Stud. **75**, 201-228. (10.1111/j.1467-937X.2007.00469.x)

[68] Rabin M. 1994 Cognitive dissonance and social change. J. Econ. Behav. Organ. **24**, 177-194. (10.1016/0167-2681(94)90066-3)

[69] Zino L, Ye M, Cao M. 2020 A two-layer model for coevolving opinion dynamics and collective decision-making in complex social systems. Chaos **20**, 083107. (10.1063/5.0004787) 32872837

[70] Centola D, Willer R, Macy M. 2005 The emperor’s dilemma: a computational model of self-enforcing norms. AJS **110**, 1009-1040. (10.1086/427321)

[71] Friedkin NE, Proskurnikov AV, Tempo R, Parsegov SE. 2016 Network science on belief system dynamics under logic constraints. Science **354**, 321-326. (10.1126/science.aag2624) 27846564

[72] Redner S. 2019 Reality inspired voter models: a mini-review. C. R. Phys. **20**, 275-292. (10.1016/j.crhy.2019.05.004)

[73] Watts DJ. 2002 A simple model of global cascades on random networks. Proc. Natl Acad. Sci. USA **99**, 5766-5771. (10.1073/pnas.082090499) 16578874 PMC122850

[74] Azar O. 2004 What sustains social norms and how they evolve? The case of tipping. J. Econ. Behav. Organ. **54**, 49-64. (10.1016/j.jebo.2003.06.001)

[75] Azar OH. 2008 Evolution of social norms with heterogeneous preferences: a general model and an application to the academic review process. J. Econ. Behav. Organ. **65**, 420-435. (10.1016/j.jebo.2006.03.006)

[76] Gavrilets S. 2021 Coevolution of actions, personal norms and beliefs about others in social dilemmas. Evol. Hum. Sci. **3**, e44. (10.1017/ehs.2021.40) PMC10427329 37588544

[77] Gavrilets S, Richerson J. 2017 Collective action and the evolution of social norm internalization. Proc. Natl Acad. Sci. USA **114**, 6068-6073. (10.1073/pnas.1703857114) 28533363 PMC5468620

[78] Tverskoi D, Guido A, Andrighetto G, Sanchez A, Gavrilets S. 2021 Disentangling material, social, and cognitive determinants of human behavior and beliefs. SocArXiv. (10.31235/osf.io/z5m9h)

[79] Argote L, Epple D. 1990 Learning curves in manufacturing. Science **247**, 920-924. (10.1126/science.247.4945.920) 17776451

[80] Arrow K. 1962 The economic implications of learning by doing. Rev. Econ. Stud. **29**, 155-173. (10.2307/2295952)

[81] Fudenberg D, Tirole J. 1983 Learning-by-doing and market performance. Bell J. Econ. **14**, 522-530. (10.2307/3003653)

[82] Lin Y, Han X, Wang B. 2014 Dynamics of human innovative behaviors. Physica A **394**, 74-81. (10.1016/j.physa.2013.09.039)

[83] Young H. 2009 Innovation diffusion in heterogeneous populations: contagion, social influence, and social learning. Am. Econ. Rev. **99**, 1899-1924. (10.1257/aer.99.5.1899)

[84] Geroski P. 2000 Models of technology diffusion. Res. Policy **29**, 603-625. (10.1016/S0048-7333(99)00092-X)

[85] Anzanello M, Fogliatto F. 2011 Learning curve models and applications: literature review and research directions. Int. J. Ind. Ergon. **41**, 573-583. (10.1016/j.ergon.2011.05.001)

[86] Davidovitch L, Parush A, Shtub A. 2008 Simulation-based learning: the learning–forgetting–relearning process and impact of learning history. Comput. Educ. **50**, 866-880. (10.1016/j.compedu.2006.09.003)

[87] Eden C, Williams T, Ackermann F. 1998 Dismantling the learning curve: the role of disruptions on the planning of development projects. Int. J. Prod. Manage. **16**, 131-138. (10.1016/S0263-7863(97)00053-7)

[88] Jaber M, Bonney M. 1996 Production breaks and the learning curve: the forgetting phenomenon. Appl. Math. Modell. **20**, 162-169. (10.1016/0307-904X(95)00157-F)

[89] Jaber M, Bonney M. 2007 Economic manufacture quantity (EMQ) model with lot-size dependent learning and forgetting rates. Int. J. Prod. Econ. **108**, 359-367. (10.1016/j.ijpe.2006.12.020)

[90] Jaber M, Kher H. 2004 Variant versus invariant time to total forgetting: the learn–forget curve model revisited. Comput. Ind. Eng. **46**, 697-705. (10.1016/j.cie.2004.05.006)

[91] Yelle L. 1979 The learning curve: historical review and comprehensive survey. Decis. Sci. **10**, 302-328. (10.1111/j.1540-5915.1979.tb00026.x)

[92] Argote L. 1993 Group and organizational learning curves: individual, system and environmental components. Br. J. Soc. Psychol. **32**, 31-51. (10.1111/j.2044-8309.1993.tb00984.x)

[93] Fioretti G. 2007 The organizational learning curve. Eur. J. Oper. Res. **177**, 1375-1384. (10.1016/j.ejor.2005.04.009)

[94] Knecht G. 1974 Costing, technological growth and generalized learning curves. Oper. Res. Q. **25**, 487-491. (10.1057/jors.1974.82)

[95] Towill D. 1990 Forecasting learning curves. Int. J. Forecast. **6**, 25-38. (10.1016/0169-2070(90)90095-S)

[96] Wright T. 1936 Factors affecting the cost of airplanes. J. Aeronaut. Sci. **3**, 122-128. (10.2514/8.155)

[97] Goeree JK, Holt CA, Palfrey TR. 2016 Quantal response equilibrium. Princeton, NJ: Princeton University Press.

[98] Akerlof G. 1980 A theory of social custom, of which unemployment may be one consequence. Q. J. Econ. **94**, 749-775. (10.2307/1885667)

[99] Bernheim B. 1994 A theory of conformity. J. Pol. Econ. **102**, 841-877. (10.1086/261957)

[100] Górges L, Nosenzo D. 2020 Measuring social norms in economics: why it is important and how it is done. Anal. Kritik **42**, 285-311. (10.1515/auk-2020-0012)

[101] Loewenstein G, Molnar A. 2018 The renaissance of belief-based utility in economics. Nat. Hum. Behav. **2**, 166-167. (10.1038/s41562-018-0301-z)

[102] Nyborg K. 2018 Social norms and the environment. Annu. Rev. Resour. Econ. **10**, 405-423. (10.1146/annurev-resource-100517-023232)

[103] Gavrilets S, Shrestha M. 2021 Evolving institutions for collective action by selective imitation and self-interested design. Evol. Hum. Behav. **42**, 1-11. (10.1016/j.evolhumbehav.2020.05.007)

[104] Perry L, Gavrilets S. 2020 Foresight in a game of leadership. Soc. Res. **10**, 1-10. 10.1038/s41598-020-57562-1 PMC7010814 32041963

[105] Perry L, Shrestha M, Vose M, Gavrilets S. 2018 Collective action problem in heterogeneous groups with punishment and foresight. J. Stat. Phys. **172**, 293-312. (10.1007/s10955-018-2012-2)

[106] Szpunar KK, Spreng RN, Schactera DL. 2014 A taxonomy of prospection: introducing an organizational framework for future-oriented cognition. Proc. Natl Acad. Sci. USA **111**, 18 414-18 421. (10.1073/pnas.1417144111) PMC4284580 25416592

[107] Berns GS, Laibson D, Loewenstein G. 2007 Intertemporal choice—toward an integrative framework. Trends Cogn. Sci. **11**, 482-488. (10.1016/j.tics.2007.08.011) 17980645

[108] Frederick S, Loewenstein G, O’Donoghue T. 2002 Time discounting and time preference: a critical review. J. Econ. Lit. **40**, 351-401. (10.1257/jel.40.2.351)

[109] Festinger L. 1957 A theory of cognitive dissonance, vol. 2. Stanford, CA: Stanford University Press.

[110] Song G, Ma Q, Wu F, Li L. 2012 The psychological explanation of conformity. Soc. Behav. Pers.: Int. J. **40**, 1365-1372. (10.2224/sbp.2012.40.8.1365)

[111] Cialdini RB, Goldstein NJ. 2004 Social influence: compliance and conformity. Annu. Rev. Psychol. **55**, 591-621. (10.1146/annurev.psych.55.090902.142015) 14744228

[112] Akerlof GA. 1997 Social distance and social decisions. Econometrica **65**, 1005-1027. (10.2307/2171877)

[113] Fehr E, Schurtenberger I. 2018 Normative foundations of human cooperation. Nat. Hum. Behav. **2**, 458-468. (10.1038/s41562-018-0385-5) 31097815

[114] Fershtman C, Weiss Y. 1998 Social rewards, externalities and stable preferences. J. Public Econ. **70**, 53-73. (10.1016/S0047-2727(98)00060-7)

[115] Kandel E, Lazear EP. 1992 Peer pressure and partnerships. J. Polit. Econ. **100**, 801-817. (10.1086/261840)

[116] Galesic M, Stein DL. 2019 Statistical physics models of belief dynamics: theory and empirical tests. Physica A **519**, 275-294. (10.1016/j.physa.2018.12.011)

[117] Gavrilets YN. 2003 Stochastic modeling of between-group social interactions. Econ. Math. Methods **39**, 106-116. [in Russian.]

[118] Kashima Y, Perfors A, Ferdinand V, Pattenden E. 2021 Ideology, communication and polarization. Phil. Trans. R. Soc. B **376**, 20200133. (10.1098/rstb.2020.0133) 33612005 PMC7935022

[119] De Vita J. 1979 Niche separation and the broken-stick model. Am. Nat. **114**, 171-178. (10.1086/283466)

[120] MacArthur R. 1957 On the relative abundance of bird species. Proc. Natl Acad. Sci. USA **43**, 293-295. (10.1073/pnas.43.3.293) 16590018 PMC528435

[121] Stoneman P, Diederen P. 1994 Technology diffusion and public policy. Econ. J. **104**, 918-930. (10.2307/2234987)

[122] Heine S, Lehman D. 1997 Culture, dissonance, and self-affirmation. Pers. Soc. Psychol. Bull. **23**, 389-400. (10.1177/0146167297234005)

[123] Henrich J. 2015 The secret of our success. Princeton, NJ: Princeton University Press.

[124] Richerson PJ, Gavrilets S, de Waal FBM. 2021 Modern theories of human evolution foreshadowed by Darwin’s *Descent of Man*. Science **372**, eaba3776. (10.1126/science.aba3776) 34016754

[125] Granovetter M. 1978 Threshold models of collective behavior. AJS **83**, 1420-1443.

[126] Rashevsky N. 1949 Mathematical biology of social behavior. III. Bull. Math. Biol. **11**, 255-271. 10.1007/BF02477979 15394463

[127] Rashevsky N. 1951 Mathematical biology of social behavior. Chicago, IL: University of Chicago Press.

[128] Rashevsky N. 1965 A note on imitative behavior. Bull. Math. Biophys. **27**, 311-313. (10.1007/BF02478408) 5866999

[129] Rashevsky N. 1965 On imitative behavior. Bull. Math. Biophys. **27**, 175-185. (10.1007/BF02498773) 5832696

[130] Davis R, Campbell R, Hildon Z, Hobbs L, Michie S. 2015 Theories of behaviour and behaviour change across the social and behavioural sciences: a scoping review. Health Psychol. Rev. **9**, 323-344. (10.1080/17437199.2014.941722) 25104107 PMC4566873

[131] Martins A. 2008 Continuous opinions and discrete actions in opinion dynamics problems. Int. J. Mod. Phys. C **19**, 617-624. (10.1142/S0129183108012339)

[132] Martins A. 2008 Mobility and social network effects on extremist opinions. Phys. Rev. E **78**, 036104. (10.1103/PhysRevE.78.036104) 18851102

[133] Jager W, Janssen M, De Vries H, De Greef J, Vlek C. 2000 Behaviour in commons dilemmas: *Homo economicus* and *Homo psychologicus* in an ecological-economic model. Ecol. Econ. **35**, 357-379. (10.1016/S0921-8009(00)00220-2)

[134] Schwarz N, Ernst A. 2009 Agent-based modeling of the diffusion of environmental innovations—an empirical approach. Technol. Forecast. Soc. Change **76**, 497-511. (10.1016/j.techfore.2008.03.024)

[135] Kaufmann P, Stagl S, Franks D. 2009 Simulating the diffusion of organic farming practices in two New EU Member States. Ecol. Econ. **68**, 2580-2593. (10.1016/j.ecolecon.2009.04.001)

[136] Zhang T, Nuttall W. 2011 Evaluating government’s policies on promoting smart metering diffusion in retail electricity markets via agent-based simulation. J. Prod. Innov. Manage. **28**, 169-186. (10.1111/j.1540-5885.2011.00790.x)

[137] Ajzen I. 1991 The theory of planned behavior. Organ. Behav. Hum. Decis. Process. **50**, 179-211. (10.1016/0749-5978(91)90020-T)

[138] Bakar MFA, Talukder M, Quazi A, Khan I. 2020 Adoption of sustainable technology in the Malaysian SMEs sector: does the role of government matter? Information **11**, 215. (10.3390/info11040215)

[139] Delre S, Jager W, Janssen M. 2007 Diffusion dynamics in small-world networks with heterogeneous consumers. Comput. Math. Organ. Theory **13**, 185-202. (10.1007/s10588-006-9007-2)

[140] Dodson Jr J, Muller E. 1978 Models of new product diffusion through advertising and word-of-mouth. Manage. Sci. **24**, 1568-1578. (10.1287/mnsc.24.15.1568)

[141] Goldenberg J, Libai B, Muller E. 2001 Talk of the network: a complex systems look at the underlying process of word-of-mouth. Mark. Lett. **12**, 211-223. (10.1023/A:1011122126881)

[142] Goldenberg J, Libai B, Moldovan S, Muller E. 2007 The NPV of bad news. Int. J. Res. Mark. **24**, 186-200. (10.1016/j.ijresmar.2007.02.003)

[143] Bernays E. 1928 Propaganda. Brooklyn, NY: Ig Publishing.

[144] Jowett GS, O’Donnell V. 2018 Propaganda & persuasion. Thousand Oaks, CA: Sage Publications.

[145] Atwood C, Griffith M, Harwell L, Schlienger E, Ensz M, Smugeresky J, Romero T, Greene D, Reckaway D. 1998 Laser engineered net shaping (lens^TM^): a tool for direct fabrication of metal parts. In *Int. Cong. on Applications of Lasers & Electro-Optics*, vol. 1998, pp. E1–E7. Laser Institute of America.

[146] Kobryn PA, Semiatin SL. 2001 Mechanical properties of laser-deposited Ti-6Al-4V. In *Int. Solid Freeform Fabrication Symp.*, pp. 179–186. See http://utw10945.utweb.utexas.edu/Manuscripts/2001/2001-21-Kobryn.pdf.

[147] Gibson I, Rosen D, Stucker B. 2015 Additive manufacturing technologies: 3D printing, rapid prototyping and direct digital manufacturing. 2nd edn. Berlin, Germany: Springer.

[148] The White House Office of the Press Secretary. 2011 President Obama launches advanced manufacturing partnership. See https://obamawhitehouse.archives.gov/the-press-office/2011/06/24/president-obama-launches-advanced-manufacturing-partnership.

[149] The White House Office of the Press Secretary. 2012 We can’t wait: Obama administration announces new public-private partnership to support. See https://obamawhitehouse.archives.gov/the-press-office/2012/08/16/we-can-t-wait-obama-administration-announces-new-public-private-partners.

[150] DebRoy T et al. 2018 Additive manufacturing of metallic components—process, structure and properties. Prog. Mater. Sci. **92**, 112-224. (10.1016/j.pmatsci.2017.10.001)

[151] Kitayama S, Snibbe A, Markus H, Suzuki T. 2004 Is there any ‘free’ choice? Self and dissonance in two cultures. Psychol. Sci. **15**, 527-533. (10.1111/j.0956-7976.2004.00714.x) 15270997

[152] Snibbe A, Markus H. 2005 You can’t always get what you want: educational attainment, agency, and choice. J. Pers. Soc. Psychol. **88**, 703-720. (10.1037/0022-3514.88.4.703) 15796669

[153] Yates J, de Oliveira S. 2016 Culture and decision making. Organ. Behav. Hum. Decis. Process **136**, 106-118. (10.1016/j.obhdp.2016.05.003) 32288179 PMC7126161

[154] Rieger MO, Wang M, Hens T. 2021 Universal time preference. PLoS ONE **16**, e0245692. (10.1371/journal.pone.0245692) 33596234 PMC7888607

[155] Strathman A, Gleicher F, Boninger D, Edwards C. 1994 The consideration of future consequences: weighing immediate and distant outcomes of behavior. J. Pers. Soc. Psychol. **66**, 742-752. (10.1037/0022-3514.66.4.742)

[156] Tan C, Johnson R. 1996 To wait or not to wait: the influence of culture on discounting behavior. In Perspectives on judgment and decision making (ed. W Loke), pp. 297-305. Lanham, MD: Scarecrow Press.

[157] Du W, Green L, Myerson J. 2002 Cross-cultural comparisons on discounting delayed and probabilistic rewards. Psychol. Rec. **52**, 479-492. (10.1007/BF03395199)

[158] Mahajna A, Benzion U, Bogaire R, Shavit T. 2008 Subjective discount rates among Israeli Arabs and Israeli Jews. J. Socio-Econ. **37**, 2513-2522. (10.1016/j.socec.2007.11.003)

[159] Wang M, Rieger M, Hens T. 2016 How time preferences differ: evidence from 53 countries. J. Econ. Psychol. **52**, 115-135. (10.1016/j.joep.2015.12.001)

[160] Fu P et al. 2004 The impact of societal cultural values and individual social beliefs on the perceived effectiveness of managerial influence strategies: a meso approach. J. Int. Bus. Stud. **35**, 284-305. (10.1057/palgrave.jibs.8400090)

[161] Venaik S, Zhu Y, Brewer P. 2013 Looking into the future: Hofstede long term orientation versus GLOBE future orientation. Cross Cult. Manage.: Int. J. **20**, 361-385. (10.1108/CCM-02-2012-0014)

[162] Tian M, Deng P, Zhang Y, Salmador M. 2018 How does culture influence innovation? A systematic literature review. Manage. Decis. **56**, 1088-1107. (10.1108/MD-05-2017-0462)

[163] Naor M, Linderman K, Schroeder R. 2010 The globalization of operations in Eastern and Western countries: unpacking the relationship between national and organizational culture and its impact on manufacturing performance. J. Oper. Manage. **28**, 194-205. (10.1016/j.jom.2009.11.001)

[164] Chua R, Huang K, Jin M. 2019 Mapping cultural tightness and its links to innovation, urbanization, and happiness across 31 provinces in China. Proc. Natl Acad. Sci. USA **116**, 6720-6725. (10.1073/pnas.1815723116) 30833399 PMC6452675

[165] He M, Lee J. 2020 Social culture and innovation diffusion: a theoretically founded agent-based model. J. Evol. Econ. **30**, 1-41. (10.1007/s00191-020-00662-y)

[166] Gelfand M, Nishii L, Raver J. 2006 On the nature and importance of cultural tightness-looseness. J. Appl. Psychol. **91**, 1225-1244. (10.1037/0021-9010.91.6.1225) 17100480

[167] Gelfand M, Raver J, Nishii L, Leslie L, Lun J, Lim B. 2011 Differences between tight and loose cultures: a 33-nation study. Science **332**, 1100-1104. (10.1126/science.1197754) 21617077

[168] Harrington J, Gelfand M. 2014 Tightness-looseness across the 50 United States. Proc. Natl Acad. Sci. USA **111**, 7990-7995. (10.1073/pnas.1317937111) 24843116 PMC4050535

[169] Mathukrishna M, Henrich J. 2016 Innovation in the collective brain. Phil. Trans. R. Soc. B **371**, 20150192. (10.1098/rstb.2015.0192) 26926282 PMC4780534

[170] Ehrlich P, Levin S. 2005 The evolution of norms. PLoS Biol. **3**, e194. (10.1371/journal.pbio.0030194) 15941355 PMC1149491

[171] Haynes C, Luck M, McBurney P, Mahmoud S, Vítek T, Miles S. 2017 Engineering the emergence of norms: a review. Knowl. Eng. Rev. **32**, 1-31. (10.1017/S0269888917000169)

[172] Prentice D. 2018 Intervening to change social norms: when does it work? Soc. Res. **85**, 115-139. (10.1353/sor.2018.0007)

[173] Delre S, Jager W, Bijmolt T, Janssen M. 2010 Will it spread or not? The effects of social influences and network topology on innovation diffusion. J. Product. Innov. Manage. **27**, 267-282. (10.1111/j.1540-5885.2010.00714.x)

[174] Gavrilets S, Auerbach J, van Vugt M. 2016 Convergence to consensus in heterogeneous groups and the emergence of informal leadership. Sci. Rep. **6**, 29704. (10.1038/srep29704) 27412692 PMC4944200

[175] Moldovan S, Goldenberg J. 2004 Cellular automata modeling of resistance to innovations: effects and solutions. Technol. Forecast. Soc. Change **71**, 425-442. (10.1016/S0040-1625(03)00026-X)

[176] Valente T, Davis R. 1999 Accelerating the diffusion of innovations using opinion leaders. Ann. Am. Acad. Pol. Soc. Sci. **566**, 55-67. (10.1177/000271629956600105)

[177] Delre S, Jager W, Bijmolt T, Janssen M. 2007 Targeting and timing promotional activities: an agent-based model for the takeoff of new products. J. Bus. Res. **60**, 826-835. (10.1016/j.jbusres.2007.02.002)

[178] Coccia M. 2014 Socio-cultural origins of the patterns of technological innovation: what is the likely interaction among religious culture, religious plurality and innovation? Towards a theory of socio-cultural drivers of the patterns of technological innovation. Technol. Soc. **36**, 13-25. (10.1016/j.techsoc.2013.11.002)

[179] Borgers T, Sarin R. 1997 Learning through reinforcement and replicator dynamics. J. Econ. Theory **77**, 1-14. (10.1006/jeth.1997.2319)

[180] Hirschman E. 1980 Innovativeness, novelty seeking, and consumer creativity. J. Consum. Res. **7**, 283-295. (10.1086/208816)

[181] Schweizer T. 2006 The psychology of novelty-seeking, creativity and innovation: neurocognitive aspects within a work-psychological perspective. Creat. Innov. Manage. **15**, 164-172. (10.1111/j.1467-8691.2006.00383.x)

[182] Tverskoi D, Babu S, Gavrilets S. 2022 The spread of technological innovations: effects of psychology, culture and policy interventions. Zenodo. (10.5281/zenodo.5715314) PMC9214287 35754991

[183] Tverskoi D, Babu S, Gavrilets S. 2022 The spread of technological innovations: effects of psychology, culture and policy interventions. *FigShare*. (10.6084/m9.figshare.c.6035739) PMC9214287 35754991

